# Progression of cognitive impairment in Parkinson’s disease correlates with uric acid concentration

**DOI:** 10.3389/fneur.2024.1378334

**Published:** 2024-05-30

**Authors:** Rui-Xue Zhai, Hui Yu, Han Ma, Ting-Ting Liu, Ping Zhong

**Affiliations:** Department of Neurology, Suzhou Hospital Affiliated to Anhui Medical University, Suzhou, China

**Keywords:** Parkinson’s disease, cognitive impairment, uric acid, homocysteine, correlation

## Abstract

**Introduction:**

This study assessed the relationship between the progression of Parkinson’s disease (PD) with cognitive impairment and changes in serum uric acid (UA) and homocysteine (Hcy) concentrations and explored the factors influencing PD with cognitive impairment.

**Methods:**

The study randomly selected 74 patients with PD and evaluated their cognitive function using the Montreal Cognitive Assessment Scale (MoCA). Patients with PD were divided into two subgroups: those with and without cognitive impairment. PD severity was evaluated and graded using the Hoehn and Yahr (H–Y) scale. Another 60 middle-aged and older individuals without PD during the same period were selected as a control group. Blood UA and Hcy concentrations in each group were measured to assess the relationship between PD, cognitive impairment, and changes in UA and Hcy concentrations.

**Results:**

The PD group with cognitive impairment had a lower UA concentration and higher Hcy concentration. The UA concentration was significantly higher in the early PD stages than in the middle and late stages (*P*<0.05). A significant negative relationship between MoCA scores and serum UA levels was found in patients with PD, whereas a positive relationship existed between MoCA scores and serum Hcy concentrations. Regression analysis showed that a higher UA concentration was an independent protective factor for PD with cognitive impairment, while a higher Hcy concentration was a risk factor (*P*<0.05). A serum UA concentration of 212.9 mmol/L and Hcy concentration of 13.35 mmol/L could distinguish between patients with PD with or without cognitive impairment with a sensitivity of 93.2% and specificity of 43.3%.

**Conclusion:**

PD and cognitive impairment were associated with a decrease in UA concentration; the later the H–Y stage was, the lower the UA concentration was. The increase in Hcy concentration was related to PD and its cognitive impairment, whereas it is not significantly correlated with PD progression.

## 1 Introduction

Parkinson’s disease (PD), an extrapyramidal disease, is a common neurological disorder. Its clinical manifestations include motor symptoms, such as slow movement, muscle rigidity, and static tremors; non-motor symptoms include cognitive disorders, sleep disorders, and anosmia. According to the 2005 Chinese PD Epidemiology report, the PD prevalence is 1.7% among individuals aged >65 years in China, similar to that in developed countries such as Europe and the United States (1). More than 4.9 million patients with PD are expected to be in China by 2030, accounting for half of all patients with PD worldwide (2). Uric acid (UA), a naturally occurring antioxidant in the human body, has potential neuroprotective effects on PD. Compared with patients with prodromal PD who have dopaminergic degeneration, typical patients with PD have lower serum UA levels. Since the condition progresses, UA concentration in patients with PD further decreases, indicating a significant decrease in serum UA concentration throughout the phases from the prodromal phase to clinical PD (3–5). Serum concentrations in patients with PD were significantly lower than those in healthy people, according to a previous meta-analysis (6). In recent years, there have been many studies on the relationship between UA and cognitive function. Euser et al. (7) have shown that a higher concentration of UA was associated with a lower risk of Alzheimer’s disease (AD). Methionine and cysteine metabolism produce homocysteine (Hcy), an amino acid containing a thiol group. Previous studies have shown that an increase in homocysteine concentration was associated with PD (8). To date, the association between UA and Hcy with the progression of cognitive impairment in PD is still unclear. This study investigated the relationship between the course of cognitive impairment in patients with PD and changes in serum UA and Hcy concentrations.

## 2 Materials and methods

### 2.1 Study participants

The study case group comprised 74 randomly selected patients with PD without a family history admitted to our hospital between October 2019 and October 2023, aged 51–85 years, including 40 male and 34 female individuals. The guidelines of the International Association for Parkinson’s Disease and Movement Disorders guide for establishing diagnosis in patients with PD. In total, 60 healthy participants matching sex and age, with normal cognitive function evaluation without obvious brain MRI abnormality, were selected as the control group, including 32 men and 28 women aged 52–85 years. The diagnosis was made by two trained and qualified neurologists with more than 10 years of work experience and approved by the hospital’s Ethics Committee (approval number, A2021016). The exclusion criteria were: (1) secondary Parkinson’s syndrome (common drugs, including flunarizine hydrochloride capsules, metoclopramide, and vascular diseases) or hereditary Parkinson’s syndrome (e.g., hepatolenticular degeneration, Huntington’s disease); (2) progressive supranuclear palsy, P-type multisystem atrophy, and corticobasal ganglia degeneration; (3) patients with dementia, gout, tumor, heart, liver, kidney, thyroid, or other major organ diseases, and those taking diuretics, febuxostat, folic acid, or other drugs affecting UA and Hcy metabolism. Informed consent was obtained from both groups.

### 2.2 Clinical assessment and laboratory data collection

#### 2.2.1 Clinical characteristics

The study collected data on age, sex, education level, medical history, family history of disease, smoking history, drug prescriptions, and other general medical history data of the PD and control groups. Routine biochemical, electrocardiogram, craniocerebral MRI, and other examinations were performed in all participants. Those who smoked for at least 1 year were considered to have smoked one cigarette per day, or ≥ 360 cigarettes per year, and those who had quit smoking less than 1 year previously.

#### 2.2.2 Neuropsychological assessment

Within 72 h after admission, the patients were evaluated using the Chinese version of the Montreal Cognitive Assessment Scale (MoCA). The MoCA includes multiple cognitive fields, visual space, memory, and abstraction. The total score is 30 points; ≥26 points indicates normal cognitive function; ≤25 points indicates cognitive impairment (total score for those with <12 years of education plus 1 positive bias). Physicians trained in neuropsychological testing evaluated the quality of the assessment.

#### 2.2.3 Hoehn–Yahr (H–Y) grading in patients with PD

PD severity was graded using the H–Y scale. The H-Y staging and Unified Parkinson’s Disease Rating Scale scores were performed in all participants after drug discontinuation (72 h after dopaminergic agonist discontinuation and 12 h after dopaminergic drug discontinuation). Stage 0 is asymptomatic. Stage 1 has only one side of the body affected, but the balance function is not affected. Stage 1.5 involves one side of the body and affects balance. Stage 2 involves both limbs, but the balance function is unaffected. Stage 2.5 involves both sides of the body, and the balance can be restored under the post-pull test of the standard vertebra (the post-pull test is negative). Stage 3 involves the bilateral limbs and balance and is post-pull test positive, but the patient can live independently. Stage 4 indicates significantly limited mobility but the ability to stand and walk unaided. Stage 5 patients can stay in bed without help. Among the stages, 2.5 is defined as early PD, and stages 3–5 are middle and late PD, as evaluated by clinically experienced attending physicians of the neurology department.

#### 2.2.4 Blood sampling

Fasting venous blood (5 mL) was collected from the enrolled patients the morning after admission. The supernatant was collected after centrifugation for 10 min at 3500 rpm. The concentrations of blood lipids, UA, Hcy, and other indices were determined using a Beckman AU5400 biochemical analyzer.

### 2.3 Statistics

All statistical analyses were conducted using SPSS 26.0 software (IBM Corp., Armonk, NY, United States). Measurement data with a normal distribution are represented by the mean ± standard deviation. Count data are expressed as percentages (%). To compare data between the two groups, the independent sample *t*-test was used; count data comparison was conducted using the chi-square test. Continuous skewed data are represented as median and interquartile differences; the Mann–Whitney U test was used to analyze the differences between the two groups. Pearson’s method was used to analyze the correlation between UA and HCY concentrations and MoCA scores in patients with PD. The factors influencing cognitive impairment in PD were analyzed using univariate and multivariate logistic regression. An analysis of the receiver operating characteristic curves of serum UA and HCY in persons with PD and cognitive impairment was conducted using regression analysis.

## 3 Results

**Figure 1:**
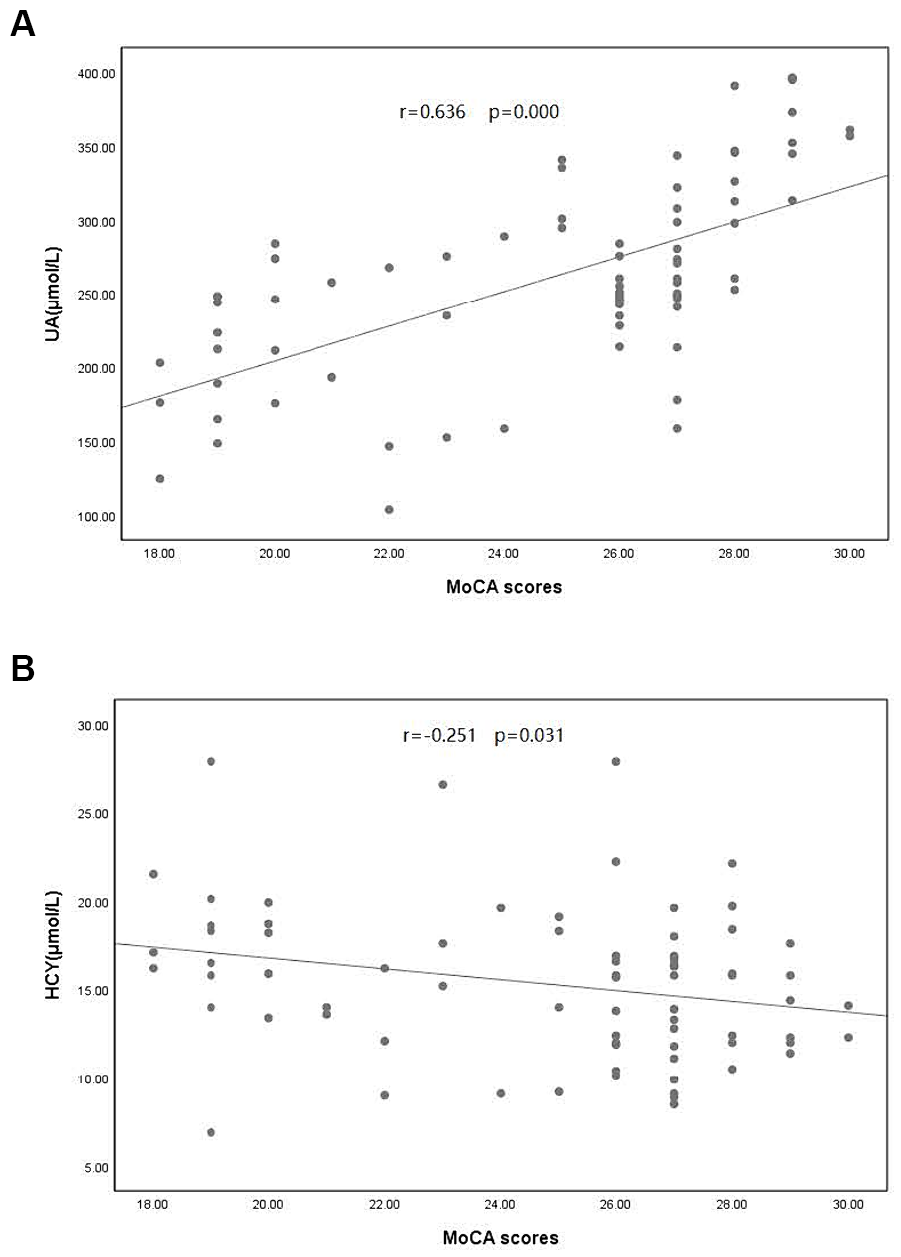
Correlation between Montreal Cognitive Assessment (MoCA) scores and **(A)** uric acid (UA) concentration and **(B)** homocysteine (Hcy) concentration in patients with PD. PD, Parkinson’s disease.

**Figure 2:**
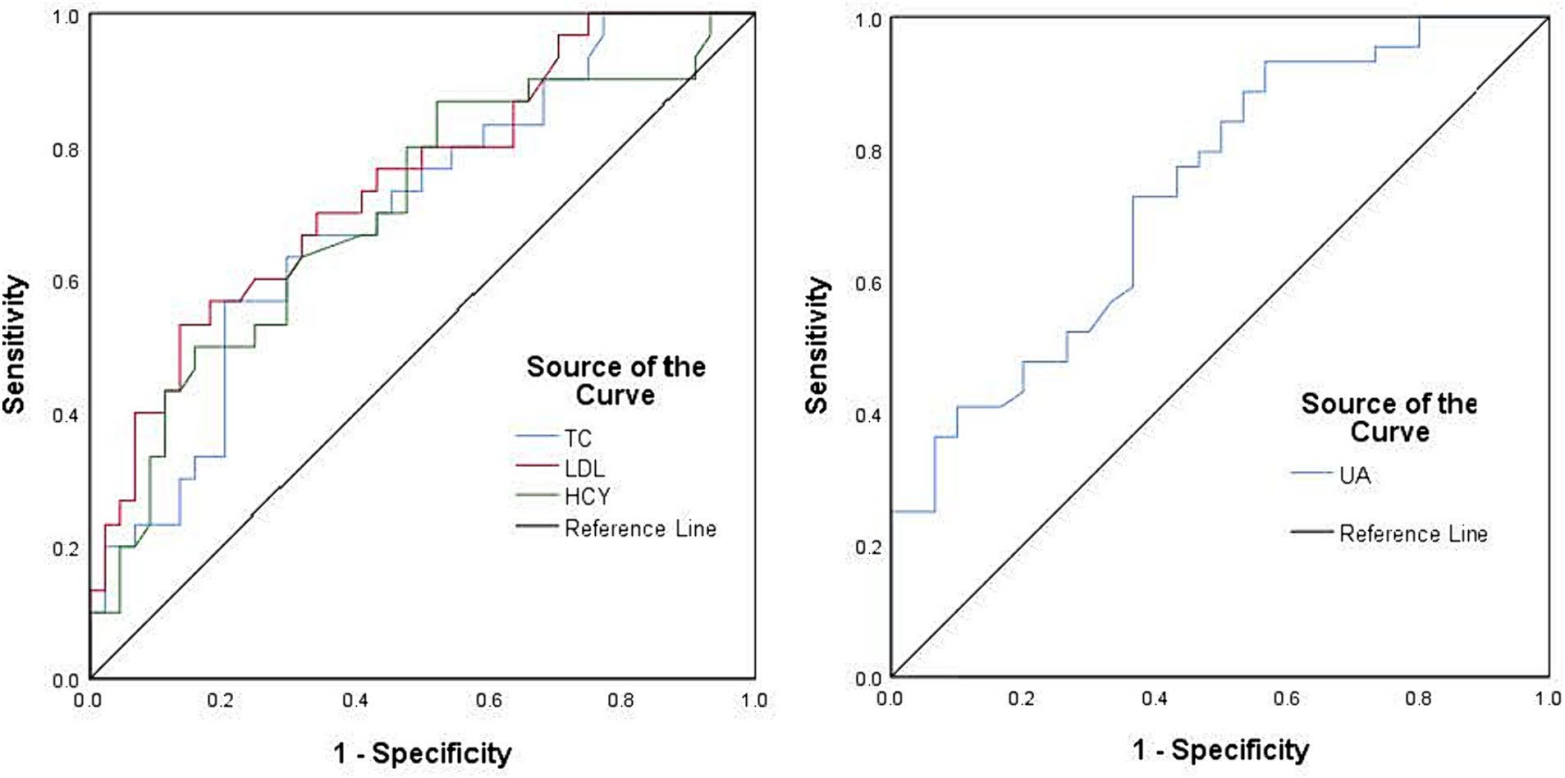
Receiver operating characteristic curves using the serum uric acid concentration (blue), homocysteine concentration (green), cholesterol concentration (blue), and low-density lipoprotein (red) were used to assess cognitive impairment in patients with Parkinson’s disease. The black line represents the ideal case.

A comparison of clinical data between the PD and control groups showed that the PD group had significantly lower years of education and a lower serum UA concentration (260.83 ± 66.69)than did the control group (311.98 ± 54.76); the Hcy concentration was significantly increased (Table 1). Compared with the subgroup without cognitive impairment, the serum UA concentration, education level, and MOCA score in the PD group with cognitive impairment decreased; the Hcy concentration, total cholesterol, and low-density lipoprotein increased significantly (Table 1). The serum UA concentration in patients with early (279.05 ± 62.50) PD was higher than in middle and late (245.34 ± 66.96) PD (Table 2). Pearson correlation analysis showed that the MoCA score of patients with PD correlated positively with blood UA concentration (*r* = 0.636, *p* = 0.000) (Figure 1A), whereas it correlated negatively with Hcy concentration (*r* = −0.251, *p* = 0.031) (Figure 1B). According to multivariate regression analysis, higher UA concentrations were associated with a lower risk of cognitive decrease in people with PD; higher Hcy concentration, cholesterol, and low-density lipoprotein were risk factors (Table 3). According to the working characteristic curve of the participants, a serum UA concentration of 212.9 mmol/L and Hcy concentration of 13.35 mmol/L could distinguish between patients with PD with or without cognitive impairment to some degree. The areas under the curves were 0.734 and 0.701, respectively, with sensitivities of 93.2 and 86.7% and specificities of 43.3 and 47.7% (Figure 2).

**Table 1:** Clinical characteristics of study participants.

Baseline data	PD group (*n* = 74)	Control group (*n* = 60)	*p*-value	PD group with cognitive impairment (*n* = 30)	PD group without cognitive impairment (*n* = 44)	*p*-value
Sex (male, %)	40 (54.05)	32 (53.33)	0.934	14 (46.67)	26 (59.1)	0.292
Age (years)	71.16 ± 7.65	69.95 ± 6.63	0.335	73.10 ± 6.790	69.84 ± 7.99	0.072
Hypertension (*n*, %)	22 (29.7)	16 (26.7)	0.696	12 (40.00)	10 (22.72)	0.111
Diabetes mellitus (*n*, %)	12 (16.2)	8 (13.3)	0.595	7 (23.33)	5 (11.36)	0.294
Smoking (*n*, %)	27 (36.5)	21 (35.0)	0.858	12 (40.00)	15 (34.09)	0.604
Education level (years)	6.38 ± 3.67	8.45 ± 3.55	0.001	5.12 ± 3.64	7.23 ± 3.47	0.014
BMI	23.2 ± 3.1	23.8 ± 2.6	0.086	22.8 ± 3.2	23.6 ± 3.5	0.102
H-Y stage	1 (1–4)	–	–	2 (1–5)	1 (1–2)	0.001
RBD	1 (0–2)	–	–	1 (1–3)	1 (0–2)	0.059
LEDD (mg)	750 (375–1,000)	–	–	875 (375–1,120)	625 (250–875)	0.413
TC (mmol/L)	4.16 ± 1.12	3.77 ± 1.04	0.623	4.63 ± 0.98	3.83 ± 1.11	0.002
TG (mmol/L)	1.49 ± 0.83	1.52 ± 0.87	0.875	1.64 ± 0.87	1.38 ± 0.79	0.207
HDL (mmol/L)	1.14 ± 0.44	1.11 ± 0.31	0.661	1.14 ± 0.44	1.13 ± 0.44	0.842
LDL (mmol/L)	2.31 ± 0.86	1.99 ± 0.92	0.628	2.76 ± 0.86	2.01 ± 0.72	0.000
UA (μmol/L)	260.83 ± 66.69	311.98 ± 54.76	0.000	227.36 ± 61.37	283.42 ± 59.84	0.000
Hcy (μmol/L)	15.32 ± 4.33	10.16 ± 3.13	0.000	17.12 ± 4.77	14.10 ± 3.55	0.003
MoCA	–	27.65 ± 0.96	–	18.19 ± 1.42	26.35 ± 0.98	0.000
MDS-UPDRS III	26.74 ± 6.49	–	–	30.2 ± 5.54	24.52 ± 6.72	0.012
MDS-UPDRS total	66.89 ± 12.38	–	–	70.21 ± 13.38	64.63 ± 11.25	0.056

BMI, body mass index; H-Y stage, Hoehn-Yahr stage; RBD, rapid eye movement sleep behavior disorder; LEDD, levodopa equivalent daily dose; TC, total cholesterol; TG, triglyceride; HDL, high-density fat eggs; LDL, low density lipoprotein; MoCA, Montreal Cognitive Assessment; UA, uric acid; Hcy, homocysteine; MDS-UPDRS, movement disorder society unified Parkinson’s Disease rating scale; PD, Parkinson’s disease.

**Table 2:** Blood UA and Hcy concentrations in early vs. middle and late stages of PD.

Group	Cases	UA (μmol/L)	Hcy (μmol/L)
H–Y 1–2.5 (early PD)	34	279.05 ± 62.50	14.37 ± 4.19
H–Y 3–5 (middle and late PD)	40	245.34 ± 66.96	16.13 ± 4.32
*T*-value	–	2.225	−1.769
*p-*value	–	0.029	0.081

H–Y, Hoehn and Yahr stage; PD, Parkinson’s disease; UA, uric acid; Hcy, homocysteine.

**Table 3:** Analysis of influencing factors of PD with cognitive impairment.

Variables	Univariate analysis			Multivariate analysis		
	OR	95%CI	*p*-value	OR	95%CI	*p*-value
Years of education (years)	0.948	0.833–1.080	0.424	0.934	0.763 ~ 1.143	0.506
BMI	0.939	0.815 ~ 1.081	0.379	0.916	0.706 ~ 1.188	0.508
H-Y stage	1.941	1.295 ~ 2.910	0.001^*^	2.021	1.140 ~ 3.583	0.016^*^
TC (mmol/L)	2.090	1.262 ~ 3.460	0.004^*^	3.229	1.315 ~ 7.926	0.011^*^
LDL (mmol/L)	3.487	1.696 ~ 7.167	0.001^*^	3.069	1.147 ~ 8.214	0.026^*^
UA (umol/L)	0.985	0.975 ~ 0.994	0.001^*^	0.983	0.968 ~ 0.997	0.020^*^
Hcy (umol/L)	1.203	1.054 ~ 1.372	0.006^*^	1.195	0.993 ~ 1.438	0.041^*^
MoCA	0.313	0.172 ~ 0.572	0.000^*^	0.142	0.032 ~ 0.629	0.010^*^

BMI, body mass index; H-Y stage, Hoehn-Yahr stage; TC, total cholesterol; LDL, low-density lipoprotein; MoCA, Montreal Cognitive Assessment; UA, uric acid; Hcy, homocysteine; OR, odds ratio; CI, confidence interval ^*^p < 0.05.

## 4 Discussion

One major pathological change in PD is the degeneration and death of dopaminergic nerve cells in the midbrain substantia nigra. However, the exact cause and pathogenesis of neuronal degeneration and death remain unclear. The degeneration of dopaminergic neurons may be caused by multiple mechanisms, including oxidative stress, free radical production, inflammation or immune responses, and apoptosis, influenced by multiple factors (e.g., genetics and the environment). Cognitive impairment is a non-motor symptom of PD. A cross-sectional study of patients with PD showed that approximately 29.1% had mild cognitive impairment, and 32.1% progressed to dementia as the disease developed. Therefore, routine cognitive assessments are recommended in patients with PD (9). Epidemiology shows that the prevalence of mild cognitive impairment in patients with PD is 20–40%, depending on the composition of the studied population. The incidence rate of dementia in patients with PD increases with the disease duration, from 30% in 5 years to >80% after 20 years (10). Studies suggest that cognitive impairment can occur during the PD prodromal phase (11). UA is an important antioxidant in the human body. It can remove free radicals, peroxides, and singlet oxygen and inhibit free radicals generation. Under normal conditions, UA reduces oxidative stress and has a protective effect on the nervous system. Serum UA concentrations decrease in patients with PD, decreasing further with disease progression (12). In the PD group, UA concentrations were lower compared with control group, according to this study; the UA concentrations were lower in middle- and late-stage PD than in the early-stage (*p* < 0.05). The pathogenesis of PD and effects of consuming UA, which has antioxidant effects, suggest that UA is a potential biomarker for PD progression. Studies on the relationship between UA and cognitive function have shown inconsistent results. A 9-year prospective cohort study showed that high blood UA levels were associated with a reduced risk of mild cognitive impairment in older Chinese people (13). High UA levels correlate negatively with cognitive function in the older adults (14). Studies also indicate that blood UA levels are not associated with the risk of cognitive impairment (15).

Recently, correlations among UA, PD, and cognitive impairment have attracted attention. A study in 2008 found that patients with PD with lower blood UA concentrations had poorer cognitive function (16). In addition, a prospective longitudinal study with a 3-year follow-up found that plasma UA concentrations remained stable during the 3-year follow-up period; the protection of neurological function decreased as PD progressed (17). Blood UA concentration significantly and negatively correlates with cognitive impairment, dysphagia, anxiety, depression, and other non-motor symptoms (18). However, the correlation between blood UA levels and cognitive impairment/dementia may vary according to the dementia subtype. Vascular dementia, AD, and PD-related dementia are not consistent with UA levels; some have protective effects on cognition, and others have harmful effects (19). Some studies have also indicated that UA concentration has no significant impact or cannot be determined on the risk of dementia in patients with PD (20, 21). Our study showed that the UA concentration was significantly lower in patients with PD with cognitive impairment compared with those without. Binary regression analysis revealed that a greater UA concentration was a protective factor in patients with PD and cognitive impairment; preliminary findings suggest that a lower UA concentration may be a risk factor for cognitive impairment in patients with PD. The possible mechanisms are as follows: First, as the concentration of UA decreases, the rate of oxidative stress response and oxidative damage accelerates, thereby accelerating the occurrence and development of diseases (22). Second, UA can enhance the activity of superoxide dismutase and reduce harmful small molecule substances production such as peroxyl radicals, hydroxyl radicals, and free radicals produced by the decomposition of peroxynitrite, thereby protecting nerve cells (23). Reduce neuronal apoptosis by inhibiting lipid peroxidation and reducing the accumulation of reactive oxygen species. Fourth, UA prevents amyloid-induced neuronal apoptosis and cholinergic dysfunction through antioxidant effects, reduces the toxic effects of amyloid on neurons, and thus protects neurons. Numerous studies have shown that UA has neuroprotective effects on PD. However, some studies have contradicted this conclusion. The latest neuroprotective trial to increase UA concentration in patients with PD showed no significant difference in clinical progression compared to the placebo group (24).

As early as 1969, researchers proposed that Hcy could cause vascular endothelial cell damage, promote lipid oxidation, and be related to atherosclerosis (25). Tinelli et al. (26) showed that prolonged exposure to high-Hcy environments can lead to cardiovascular and neurodegenerative diseases, including AD and PD. An increased blood Hcy concentration is a risk factor for dementia in the general population. Levodopa treatment for PD increases the blood Hcy concentration; however, whether this increase is related to cognitive impairment is unclear in patients with PD. A series of studies have shown that higher serum HCY concentration is associated with cognitive dysfunction in patients with PD and may be an intervenable risk factor for cognitive decline in these patients (27, 28).

Tchantchou et al. (29) showed that high Hcy levels induce oxidative stress and cortical damage in rats, affecting cognition. Cognitive damage occurs when Hcy is >15 μmol/L, as confirmed by Sampedro et al. (30). Our study showed that the average concentration of Hcy was significantly greater in the PD group (15.32 ± 4.33 μmol/L) than in the control group. The Hcy concentration was also significantly greater in the PD subgroup with than in that without cognitive impairment, consistent with the above research conclusions. Hcy concentrations in early and late stages of PD did not differ significantly. Sleeman et al. (31) showed that the increase in serum HCY concentration was correlated with the increase in motor function and ecline in cognitive function in patients with PD. These results suggest that Hcy concentration is not related to the progression of PD but is associated with cognitive impairment in patients with PD. Binary logistic regression analysis showed that a greater concentration of Hcy was an independent risk factor for cognitive impairment associated with PD. According to Periñán et al. (32), although the increase in Hcy concentration is a risk factor for decreased cognitive function in patients with PD, no association has been found between polymorphisms of Hcy metabolism-related genes and patients with PD with cognitive impairment. Therefore, large-scale studies on different ethnic groups are needed to evaluate the correlation between them (32).

Previous studies have shown that hypercholesterolemia was associated with decreased cognitive function (33), which is consistent with the results of this study: the cholesterol levels were significantly higher in the PD group with cognitive impairment than in that without. Hyperglycemia, hypertension, and low education level are risk factors for cognitive impairment. This study found that low education level was a risk factor for PD with cognitive impairment, whereas hypertension, diabetes, and other factors were not related to PD with cognitive impairment and might be related to the matching of the two groups of indicators and the small sample size.

The present study has a few limitations. The results from this study may have been biased because it was conducted in only one center with a small sample of participants. We plan to expand the sample size and conduct multicenter and longitudinal follow-up studies to clarify the correlation between UA and Hcy in patients with PD with cognitive impairment and identify a concentration more conducive to protecting patients with PD with cognitive impairment. This study provides new insights into diagnosing and treating PD with cognitive impairment. In addition, as this study is a single-center study, most of the enrolled patients were from the local area, and UA was measured in a population with confirmed PD. Therefore, the concentration of UA may have been affected by geographical differences, dietary habits, medication treatment, lack of exercise, constipation, and changes in gut microbiota. Therefore, the possibility of PD-related factors leading to decreased UA concentration cannot be ruled out. Further research is necessary for determining whether the decreased UA concentration is a cause or result.

## 5 Conclusion

Our study results indicated that decreased blood UA concentrations were associated with cognitive impairment and disease progression. A greater blood UA concentration was an independent protective factor for PD with cognitive impairment; a greater blood Hcy concentration was an independent risk factor. By matching risk factors such as age and family history, the correlation between blood UA and Hcy concentrations and the disease course of PD cognitive impairment was studied, finding that blood UA concentrations decrease as PD progresses toward the late H–Y stage; the Hcy concentration did not correlate with PD progression. Improving prognosis in patients with PD and cognitive impairment by maintaining UA and Hcy at appropriate concentrations might be possible.

## Data availability statement

The original contributions presented in the study are included in the article/supplementary material, further inquiries can be directed to the corresponding authors.

## Ethics statement

The studies involving humans were approved by Ethics Committee of Suzhou Municipal Hospital (approval number A2021016). The studies were conducted in accordance with the local legislation and institutional requirements. The participants provided their written informed consent to participate in this study.

## Author contributions

R-XZ: Writing – original draft, Writing – review & editing, Conceptualization, Data curation, Formal analysis, Investigation, Methodology, Software, Supervision. HY: Formal analysis, Methodology, Writing – review & editing. HM: Data curation, Investigation, Writing – review & editing. T-TL: Data curation, Investigation, Writing – review & editing. PZ: Formal analysis, Methodology, Resources, Writing – review & editing.
